# 

**Published:** 1900-09

**Authors:** 


### TWENTY-SIXTH ANNUAL MEETING MISSISSIPPI VALLEY MEDICAL ASSOCIATION, ASHEVILLE, N. C., OCTOBER 9, 10, 11, 1900.

OFFICE OF THE SECRETARY, NO. 11 WEST KENTUCKY ST.

LOUISVILLE, KY., July 6, 1900.—DEAR DOCTOR: Your attention is called to the twenty-sixth annual meeting of the Mississippi Valley Medical Association, which will be held Tuesday, Wednesday and Thursday, October 9, 10, 11 next, under the presidency of Dr. Harold N. Moyer, of Chicago. At a meeting of the executive committee held at Atlantic City, June 6th, the following were chosen to deliver the annual addresses: Dr. I. N. Love, of St. Louis, the address in Medicine; Dr. C. A. Wheaton, of St. Paul, Minn., the address in Surgery. The mere mention of these names is guarantee sufficient that the Association will hear only the best.

Negotiations are in progress by which the members of the Association may obtain a one-fare rate for the round trip for this meeting. The Southeastern Passenger Association has already granted this rate, and it is believed that the Western and Central Passenger Associations will concur. Due notice of this will be sent later.

It is earnestly requested that those desiring to read papers will send their titles to the secretary within the next thirty days. It is also requested that a brief synopsis of the paper of not less than twenty-five words accompany the title.

The Association will not be divided into sections at this meeting. The headquarters will be at the Battery Park Hotel, at which place the sessions will be held.

We trust you may be able to attend and read a paper at the next meeting, which promises to be a most enthusiastic and successful one.

Those who will read papers are requested to hand them to the secretary—typewritten—for use of the publication committee.

Fraternally yours,

HENRY E. TULEY, Secretary.

---

COLLEGE OF PHYSICIANS.—The will of the late Dr. John Ashhurst, Jr., provides for a handsome addition to the library of this institution. The college is to have its selection from his books of a number not exceeding 1,500 volumes.

---

***NOTICE.—This JOURNAL and THE INTERNATIONAL JOURNAL OF SURGERY one year for $1.00 cash, to new subscribers. No out-of-town checks accepted unless 10c. is added for cost of cashing. Strictly to new subscribers.***

### THE TRI-STATE MEDICAL SOCIETY OF ALABAMA, GEORGIA AND TENNESSEE.

WE desire to call the attention of our readers to the next meeting of the Tri-State Medical Society at Chattanooga, October 11, 12 and 13, 1900.

This is an active, working society, growing from year to year, and is destined to accomplish great good for the profession in the States which it represents. Every member of the profession in Alabama, Tennessee and Georgia should take an active interest in this association, become members, attend its meetings and lend their influence in building it up to the high standard which it should attain.

This gives us an opportunity each year of meeting and interchanging views with the leading members of the profession in three States. It also gives us an opportunity of meeting our professional neighbors and becoming better acquainted with them in a social way.

The annual dues are small, the length of meeting short, so that it requires but little loss of time to attend, thus getting a short respite from work, and at the same time the beneficial influence of hearing papers read and discussed from various standpoints.

This society gathers each year leading general practitioners, surgeons, gynecologists and the various specialists from the three States which it represents besides the visitors, and each subject is discussed on a broad basis from different views, which makes the meeting more generally useful, interesting and of greater benefit to all.

With a little more enthusiasm and interest from the profession the Tri-State Society can be made the leading society in this section of the country. We have the men and material for such a society, and all that is needed is for each member of the regular profession in good standing to send in their names, become members and active workers for the benefit of the profession.

All those who desire to read papers should send in titles to same at once to the Secretary, Frank Trester Smith, M.D., Chattanooga, Tenn., or to the President, R. R. Kime, Atlanta, Ga., so that their names may appear in proper place on the regular program.

From what we learn the prospects bid fair for an excellent meeting, which no one can afford to miss if they can possibly attend.

---

WE have just received a reprint of a paper published by Dr. George Foy in *Janus* with the title of "Crawford Williamson Long, the Discoverer of Ether Anesthesia." In this article Dr. Foy seeks to again establish the priority claims of Dr. Long as the first physician to use successfully ether anesthesia in surgical operations. He quotes largely and gives due credit to the admirable article of Dr. Luther B. Grandy on the same subject, published some time ago in this JOURNAL. This article shows conclusively the rightful claims of Dr. Long. Future history must certainly recognize the justness of this claim.

---

THE venerable Dr. A. W. Griggs, the Nestor of the medical profession of Georgia, is dead. Dr. Griggs was a unique figure in the profession, affable, sociable, talented and old enough to have had a world of experience. He was a constant attendant upon the meetings of the State Medical Association, and his presence will be greatly missed.

---

***NOTICE.—This JOURNAL and THE INTERNATIONAL JOURNAL OF SURGERY one year for $1.00 cash, strictly to new subscribers. No out-of-town checks accepted unless 10c. is added for cost of cashing. Strictly to new subscribers.***

**This Journal and Atlanta Semi-weekly Journal one year for $1.00 cash in advance. No checks. Strictly to new subscribers.**

**This Journal and Atlanta Semi-weekly Journal one year for $1.00 cash in advance. No checks. Strictly to new subscribers.**

